# Glucagon-like peptide 1 and glucose-dependent insulinotropic peptide hormones and novel receptor agonists protect synapses in Alzheimer’s and Parkinson’s diseases

**DOI:** 10.3389/fnsyn.2022.955258

**Published:** 2022-07-27

**Authors:** Christian Hölscher

**Affiliations:** Academy of Chinese Medical Sciences, Henan University of Chinese Medicine, Zhengzhou, China

**Keywords:** LTP, synaptic plasticity, growth factor, incretins, CNS, insulin, oxidative stress, cognition

## Abstract

Glucagon-like peptide 1 (GLP-1) and glucose-dependent insulinotropic peptide (GIP) are peptide hormones and growth factors. A major pathological feature of both Alzheimer’s dis-ease (AD) and Parkinson’s disease (PD) is the loss of synaptic transmission in the cortex in AD and the loss of dopaminergic synapses in the nigra-striatal dopaminergic projection. Several studies demonstrate that GLP-1 and GIP receptor agonists protect synapses and synaptic transmission from the toxic events that underlie AD and PD. In a range of AD animal models, treatment with GLP-1, GIP, or dual-GLP-1/GIP receptor agonists effectively protected cognition, synaptic trans-mission, long-term potentiation (LTP), and prevented the loss of synapses and neurons. In PD models, dopaminergic production resumed and synapses became functional again. Importantly, the GLP-1 receptor agonists exendin-4 and liraglutide have shown good protective effects in clinical trials in AD and PD patients. Studies show that growth factors and peptide drugs that can cross the blood–brain barrier (BBB) better are more potent than those that do not cross the BBB. We therefore developed dual-GLP-1/GIP receptor agonists that can cross the BBB at an enhanced rate and showed superior protective properties on synapses in animal models of AD and PD.

## Introduction

Alzheimer’s disease (AD) and Parkinson’s disease (PD) are the most common chronic neurodegenerative disorders. The underlying process that drives the diseases is the loss of synaptic activity, the loss of synapses, and finally, the loss of neurons (Picconi et al., 2012; Robinson et al., 2014). Both conditions are accompanied by a chronic inflammatory response of the brain. The inflammatory response further drives the diseases by increasing oxidative stress, reducing the levels of growth factors, and increasing the release of pro-inflammatory cytokines by activated microglia and astrocytes (Lee et al., 2010; Tansey and Goldberg, 2010). In AD, glutamatergic and cholinergic synapses of the temporal lobe are affected first, followed by a spread to other regions (Davies et al., 1987; Golde, 2006). In PD, the projection of the substantia nigra (SN) to the striatum appears to be one of the early manifestations of the disease, which leads to a reduction of dopamine synthesis and a failure of synaptic transmission in the striatum to activate areas that are involved in motor coordination (Picconi et al., 2005; Bagetta et al., 2010). Another key feature of the pathology of AD and PD is the reduction of growth factor signaling in the brain (Allen et al., 2013; Hölscher, 2014). Therefore, a successful strategy is to support synaptic activity, prevent the loss of synapses, and eventually, the loss of neurons, to normalize growth factor signaling and to reduce the chronic inflammatory response in the brain.

## Growth factors are neuroprotective in Alzheimer’s disease and Parkinson’s disease

The growth factor signaling mechanism is reduced in the brains of AD and PD, and growth factors have neuroprotective effects (Allen et al., 2013). Brain-derived neurotrophic factor (BDNF) can protect synapses in the hippocampus of AD mouse models. By intra-cerebroventricular (icv.) injection of BDNF, memory was improved in a spatial water maze task, the potentiation of synaptic transmission (LTP) was improved, and the number of synapses in the hippocampus was increased (Blurton-Jones et al., 2009). Increasing BDNF levels in the brain through viral gene delivery vectors showed the same neuroprotective effects. Synapse loss was reversed, and synaptic plasticity and memory formation in water maze tests was improved in mouse models of AD (Nagahara et al., 2009; Poon et al., 2009). So far, it has not been possible to develop a treatment based on BDNF, as this growth factor does not cross the blood–brain barrier (BBB). To make use of the BDNF protective effects, a method to overcome the BBB has to be developed, such as a gene delivery system to the brain, or BDNF has to be injected icv. (Schulte-Herbruggen et al., 2007; Zuccato and Cattaneo, 2009). There have been many attempts to find a solution to this problem, but none have been successful up to now. Another well-known growth factor with good neuroprotective properties is the nerve growth factor (NGF). Several studies have shown that NGF can protect synapses in the hippocampus, and improve LTP and cognition in AD mouse models or in non-primate monkeys (Clarris et al., 1994; Kordower et al., 1997; Covaceuszach et al., 2009). The same problem exists for NGF—it does not cross the BBB, and techniques and strategies must be developed to cross the BBB to develop an effective treatment for AD. Clinical trials in AD patients attempted to increase NGF production in the brain were not successful (Bradbury, 2005; Heese et al., 2006; Schulte-Herbruggen et al., 2007; Covaceuszach et al., 2009). Furthermore, clinical trials that tested gene delivery by a viral vector that had been injected into the brains of patients have also been conducted (Mueller and Flotte, 2008; Mandel, 2010; Rafii et al., 2014). Sadly, none of these approaches were successful. In PD, researchers focus on the main growth factor in treating the disease, which is the Glial cell line-derived neurotrophic factor (GDNF) (Lin et al., 1993). This growth factor has shown good effects in some animal models of PD (Kordower and Bjorklund, 2013; Migliore et al., 2014; Zhang et al., 2019). Again, the main problem for developing a drug treatment for PD is the fact that GDNF does not cross the BBB. Strategies to use GDNF gene vectors have not been successfully so far (Kells et al., 2012; Van Laar et al., 2021). Clinical trials in which GDNF was injected directly into the brain did not yield much success either (Whone et al., 2019). It is clear from these findings that while the use of growth factors to treat CNS diseases, such as AD and PD, is a promising strategy, the BBB poses a robust obstacle in turning such strategies into viable treatment options in the clinic.

### Glucagon-like peptide 1 and glucose-dependent insulinotropic peptide are growth factors that can cross the blood–brain barrier

**FIGURE 1:**
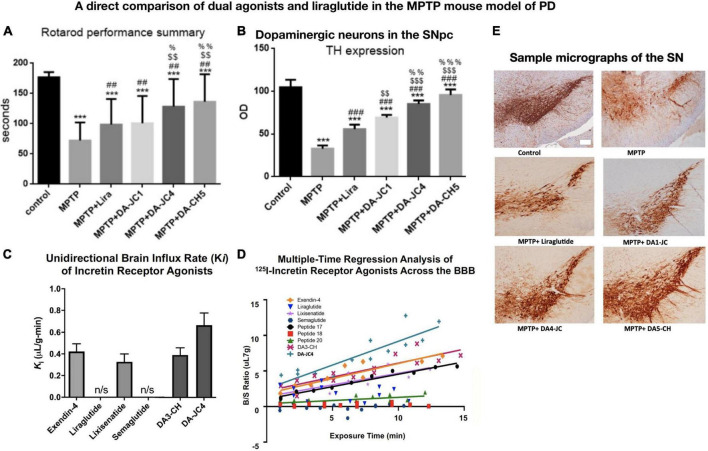
A comparison among three dual-GLP-1/GIP receptor agonists and liraglutide in the MPTP mouse model of PD, and the evaluation of BBB penetration of different peptides using ^125^I-labeled peptides. There is a direct correlation between the ability to cross the BBB and to protect the brain. **(A)** Rotarod test of motor activity. DA4-JC and DA5-CH were most protective. ***p < 0.001 compared to controls; ^##^*p* < 0.01 compared to the MPTP group; ^$$^*p* < 0.01 compared to the liraglutide + MPTP group. ^%^*p* < 0.05 compared to the DA-JC1 + MPTP group, ^%%^*p* < 0.01 compared to the DA1-JC + MPTP group. *N* = 10 per group. **(B)** Histology of dopaminergic neurons in the SN. Quantification of TH expression in neurons in the SN. ***p < 0.001 compared to control group; ^###^*p* < 0.001 compared to MPTP group; ^$$^*p* < 0.01 compared to the Liraglutide group; ^$$$^*p* < 0.001 compared to the Liraglutide group; ^%%^*p* < 0.01 compared to the DA-JC1 group; ^%%%^*p* < 0.001 compared to the DA-JC1 group. *N* = 4 per group. **(C)** Measurement of peptide influx into the brain after 10 min using ^125^I-labeled peptides. Liraglutide and semaglutide are peptides that have been acetylated with a fatty acid and show poor BBB penetration, while non-acetylated peptides can cross the BBB better. DA-JC4 can cross the BBB best **(D)** Regression analysis of influx into the brain of ^125^I-labeled peptides. **(E)** Micrographs of the SNpc, scale bar = 200 μm. **(A,B,E)** Adapted from Feng et al. (2018) and **(C,D)** adapted from Salameh et al. (2020).

GLP-1 and GIP are peptide hormones and growth factors that play important physiological signaling roles to control cell metabolism and energy utilization (Baggio and Drucker, 2007; Doyle and Egan, 2007; Finan et al., 2016). They are growth factors that can protect the brain from stressors and show good effects in animal models of AD and PD (Hölscher, 2018). GLP-1 and GIP receptors are expressed on neurons in the CNS in the cortex and other key brain areas relevant to AD and PD (Usdin et al., 1993; Merchenthaler et al., 1999; Nyberg et al., 2005; Hamilton and Hölscher, 2009; Cork et al., 2015; Graham et al., 2020). Importantly, GLP-1 and GIP can cross the BBB, as can some of the GLP-1 receptor agonists that are available in the market to treat type 2 diabetes (Kastin et al., 2002; Kastin and Akerstrom, 2003; Li et al., 2020; Salameh et al., 2020; Zhang et al., 2020). Novel dual-GLP-1/GIP receptor agonists that have been developed to treat diabetes (Finan et al., 2013) or AD and PD (Hölscher, 2018) can cross the BBB at various degrees (Kastin et al., 2002; Kastin and Akerstrom, 2003; Li et al., 2020; Salameh et al., 2020; Zhang et al., 2020). There appears to be a correlation between the ability to cross the BBB and to protect the brain. Therefore, we developed novel dual-GLP-1/GIP receptor agonists that contain cell-penetrating motifs in their amino acid sequences that can enhance BBB penetration. In a direct comparison, the dual agonists DA4-JC and DA5-CH were able to cross the BBB much better than standard GLP-1 receptor agonists, such as exendin-4, liraglutide, and semaglutide (Li et al., 2020; Salameh et al., 2020; Zhang et al., 2020). When comparing DA4-JC and DA5-CH with liraglutide or dual agonists that do not contain the cell-penetrating motifs, we found that our dual agonists were superior, and there was a clear correlation between the ability to cross the BBB and to protect the brain from toxins (Feng et al., 2018; Zhang et al., 2020). Figure 1 demonstrates the correlation between the ability to cross the BBB and the neuroprotective effects of drugs.

It is of importance to point out that drugs of the GLP-1 class that are on the market have been designed to treat diabetes. They can be given to non-diabetic people as they do not affect blood glucose and insulin levels under normoglycemic conditions (Athauda et al., 2017; Edison et al., 2020; Malatt et al., 2022). An important property of such drugs is the half-life survival in the blood. As hyperglycemia is a serious condition, drugs need to be present throughout the day to ensure blood glucose levels are within the physiological range. These drugs often have modifications, such as an addition of a fatty acid (Hedrington et al., 2018; Knudsen and Lau, 2019) or a pegylation modification (Yun et al., 2018). However, a long survival time in the blood implies slow penetration of the BBB. It follows that drugs that can enter the brain better have a shorter survival time in the blood. A recent study of BBB penetration by GLP-1 class drugs on the market and dual agonists that have modifications to cross the BBB at an enhanced rate confirmed this (Salameh et al., 2020).

## Synaptic transmission is modulated by glucagon-like peptide 1 and glucose-dependent insulinotropic peptide mimetics

GLP-1 and GIP receptor agonists are peptide hormones and growth factors, and share the neuroprotective effects of NGF and BDNF. Both GLP-1 and GIP can protect synapses and cognition. Direct injection of GLP-1 into the basal ganglia can increase the release of the neurotransmitter glutamate from neurons, demonstrating that synaptic transmitter vesicle release can be enhanced by GLP-1 (Oka et al., 1999b). In another study from this group, GLP-1 increased the spontaneous firing rate of pyramidal neurons in the hippocampus in an *in vivo* single-neuron recording set-up (Oka et al., 1999a). GLP-1, as well as GIP analogs, can enhance long-term potentiation (LTP) in the hippocampus and can protect synapses from the effects of beta-amyloid that had been injected into the brain. Agonists, such as (Val8) GLP-1, showed a clear upregulation of LTP in area CA1 of the hippocampus, while the selective GLP-1R antagonist exendin (9–36) blocked LTP. Long-acting GIP analogs that are protease-resistant showed the same enhancing effect on LTP (Gault and Hölscher, 2008a,b). The GLP-1 analog liraglutide (Victoza; Saxenda), which is currently on the market as a treatment for diabetes, showed good effects in protecting LTP and synapses in the hippocampus from the effects of icv. injected amyloid in the rat (McClean et al., 2010). In another study, liraglutide not only prevented the impairment of LTP in area CA1 in the hippocampus from amyloid icv. injections, but also protected spatial memory as shown in water maze tests (Han et al., 2013). Lixisenatide (Lyxumia and Adlyxin) is another drug that is currently on the market to treat diabetes. This drug has better BBB penetration and showed good protective effects in the same icv. amyloid injection rat model of AD. Spatial memory and hippocampal LTP were protected by lixisenatide (Cai et al., 2014). In contrast, the elimination of the GLP-1 receptor in a KO model impaired spatial learning abilities and reduced synaptic plasticity in the hippocampus (Abbas et al., 2009), as did the removal of the GIP receptor in a GIPR KO model (Faivre et al., 2011).

### Do incretins modulate synaptic transmission and vesicle release?

What could be the mechanisms that underly the effects of GLP-1 on synaptic plasticity? It has been shown that activating GLP-1 receptors on beta-cells in the pancreas enhances insulin vesicle release by a mechanism that involves the closure of K^+^ channels, which leads to the depolarization of the cell membrane and opens voltage-dependent calcium channels (VDCC). This triggers an influx of Ca^2+^ into the cell, which in turn activates Ca^2+^-sensitive enzymes, such as adenylate cyclase. This increases levels of cAMP and thereby activates protein kinase A (PKA), which then activates vesicle exocytosis mechanisms to release insulin from beta-cells (Leech and Habener, 1997; Suzuki et al., 1997; Green et al., 2004). This biochemical chain of events is identical to neuronal ones that control the release of neurotransmitter vesicles into the synaptic cleft (Winder and Conn, 1993; Wheeler et al., 1994). For example, GLP-1 modulates glutamate-induced Ca^2+^ influx in neuronal cell cultures. This effect is VDCC dependent. The influx of Ca^2+^ influx into neurons is increased by reduced K^+^ conductance, which slows membrane repolarization and enhances depolarization. GLP-1 receptor activation induces cAMP synthesis, and activates PKA, MAP kinases, and CREB (Gilman et al., 2003; Perry and Greig, 2005; Doyle and Egan, 2007). This shows that the mechanism to modulate the release of insulin vesicles is not very different from the mechanism that releases neurotransmitter vesicles at the synapse (Winder and Conn, 1993; Wheeler et al., 1994).

## Glucagon-like peptide 1 and glucose-dependent insulinotropic peptide receptor agonists protect synaptic plasticity in animal models of Alzheimer’s disease

The previous section showed that GLP-1 and GIP signaling has direct effects on synaptic transmission and LTP in the hippocampus. This indicates that receptors are located on the synapses and modulate neurotransmitter release in a similar fashion as they modulate insulin vesicle release on beta-cells in the pancreas. However, this effect is only an acute effect that is unlikely to persist when the drug has been removed. We therefore tested GLP-1 and GIP receptor agonists in animal models of AD. In a transgenic mouse model that expresses a human-mutated APP gene and a human-mutated presenilin-1 gene, APP/PS1 mice were injected subcutaneously once daily for 8 weeks. The long-acting GLP-1 analog Val(8)GLP-1 showed clear protection of hippocampal LTP in area CA1 without affecting baseline transmission in 9- and 18-months old mice. Paired-pulse facilitation, a form of local circuit plasticity that enhances the second stimulus response by reducing local GABAergic inhibition, was changed at early intervals, but not at later ones (Gengler et al., 2012). In another APP/PS1 AD model, treatment of 9 months old mice for 8 weeks once-daily ip. with liraglutide rescued memory formation and *in vivo* hippocampal LTP in area CA1. Paired-pulse facilitation had also been improved. Importantly, the expression level of the synaptic protein synaptophysin was much improved in the hippocampus and the neocortex, documenting that the synaptic loss that is common for this AD animal model has been reduced by liraglutide. In addition, the amyloid plaque load and the inflammatory response were reduced while the number of new neurons in the dentate gyrus was normalized by the drug (McClean et al., 2011). In a follow-up study, 14 months old APP/PS1 mice were treated with liraglutide once daily for 8 weeks to test if the drug had effects in animals where the amyloid-related pathology already had been established. It was found that even in aged mice, an improvement in memory formation was observed, and LTP in area CA1 was found to be strong and enduring in drug-treated AD mice but lacking in saline-treated AD mice. Synaptophysin levels were increased by the drug in both the hippocampus and the cortex (McClean and Holscher, 2014a). In a chronic study testing liraglutide, APP/PS1 mice were treated for 8 months starting at 2 months of age before the amyloid pathology developed. It was found that cognition, as well as LTP in the hippocampus, was improved, synaptophysin levels were much improved by the drug, and the key pathological AD markers were reduced. This demonstrates that treatment with liraglutide has the potential to act as a prophylactic treatment (McClean et al., 2015). On the basis of these results, we obtained funding to test liraglutide in a phase II trial in AD patients (Femminella et al., 2019, 2020; Edison et al., 2021).

**FIGURE 2:**
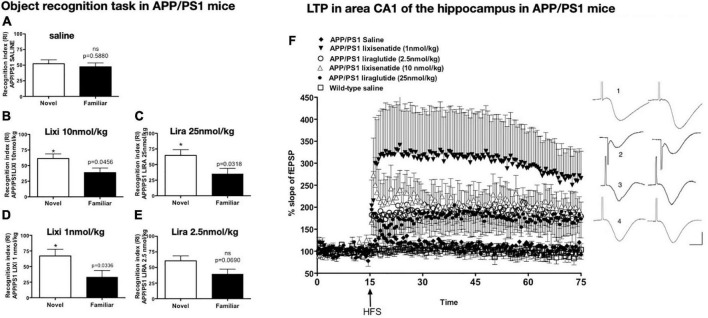
Object recognition memory after drug treatment. APP/PS1 mice failed to discriminate between novel and familiar objects while wild-type mice did not **(A)**. Treatment with lixisenatide **(B)** or liraglutide **(C)**. The low dose lixisenatide still improved memory **(D)**, but 2.5 nmol/kg liraglutide- treatment did not **(E)**. *p < 0.05 **(F)** Hippocampal LTP in area CA1 induced by weak HFS was improved after drug treatment. APP/PS1 saline compared with APP/PS1 LIXI 10 nmol/kg = (*p* < 0.0242). APP/PS1 LIXI 25 nmol/kg compared to APP/PS1 saline = (*p* < 0.005). APP/PS1 LIXI 1 nmol/kg was different from APP/PS1 LIRA 2.5 nmol/kg (*p* < 0.0148). APP/PS1 LIXI 1 nmol/kg was different from APP/PS1 saline (*p* < 0.005). APP/PS1 LIRA 2.5 nmol/kg increased LTP compared to APP/PS1 saline (*p* < 0.05). fEPSP traces are shown on the right. (1) APP/PS1 LIXI 1 nmol/kg, (2) APP/PS1 LIRA 2.5 nmol/kg, (3) wild-type, (4) APP/PS1 saline. Calibration bars vertical: 1 mV, horizontal: 10 ms. Group sizes were 8–10 animals. Figure modified from McClean and Holscher (2014b). HFS, High frequency stimulation; LTP, long-term potentiation; fEPSP, field excitatory post-synaptic potential; Lixi, lixisenatide.

The drug lixisenatide showed similar protective effects in the APP/PS1 mouse model of AD. In a dose–response comparison with liraglutide, it was found that lixisenatide was more potent in protecting memory formation, LTP in the hippocampus, and the normalization of synaptophysin levels in the hippocampus and cortex. The inflammatory response and amyloid levels were also reduced (Winder and Conn, 1993; see Figure 2).

Patch-clamp studies testing the effects of bath-applied GLP-1 demonstrated that activating the receptor in hippocampal neurons evoked acute membrane potential changes, proving that GLP-1 receptors exist in the hippocampus and can induce acute neuronal changes (Cork et al., 2015; Graham et al., 2020). In another study, amyloid_1–40_ reduced the frequency of miniature inhibitory and excitatory postsynaptic currents (mEPSCs) (mIPSCs) in rat CA1 pyramidal neurons. This shift in mEPSCs and mIPSCs was prevented with co-incubation with Val(8)GLP-1. Increases in intracellular Ca^2+^ concentrations induced by amyloid was normalized by the GLP-1 analog. These shifts were partially dependent on NMDA receptor and voltage-dependent Ca^2+^ channel activity (Wang et al., 2013).

GIP analogs showed similar neuroprotective effects in the hippocampus of APP/PS1 mice to GLP-1 mimetics. In 12 months old mice, dAla(2)GIP was given once daily for 21 days. It was found that spatial memory was rescued, and LTP in area CA1 was much improved not only in the APP/PS1 AD mouse model, but also in aged wt mice. Synapse numbers were higher in the drug group than in the saline group. The plaque load in the cortex and the chronic inflammatory response were also reduced (Faivre and Hölscher, 2013b). In a follow-up study, 19 months old mice were treated for 21 days. Synapse numbers were higher not only in the drug-treated APP/PS1 mice, but also in drug-treated litter mate wt controls. Memory formation was not significantly improved (Faivre and Hölscher, 2013a). These results demonstrate that the drug still had effects in aged APP/PS1 mice when the pathology had already manifested itself for a long time, but also in wild-type mice that showed age-related synaptic failure and numbers. The drug significantly reduced the chronic inflammatory response and oxidative stress (Duffy and Hölscher, 2013).

### Novel dual- Glucagon-like peptide 1/glucose-dependent insulinotropic peptide receptor agonists are protective in animal models of Alzheimer’s disease

**FIGURE 3:**
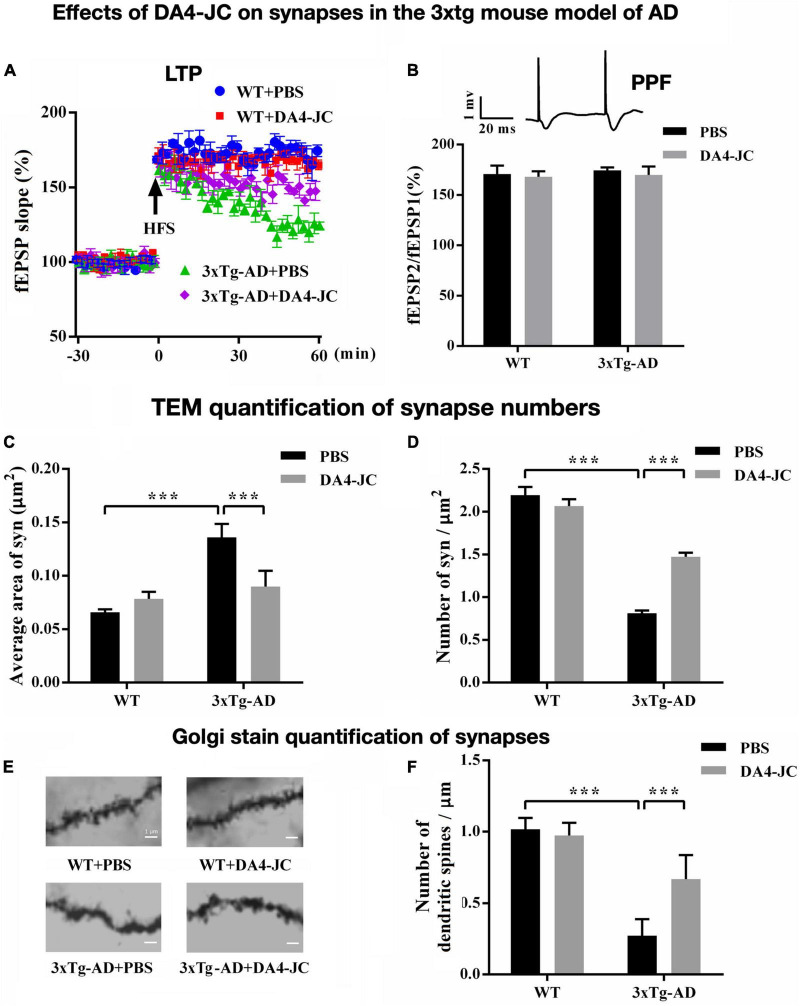
Effects of the dual agonist DA4-JC in the 3xtg mouse model of AD. **(A)** LTP is rescued by the drug in the hippocampus. **(B)** No effect was seen on paired-pulse facilitation (PPF). **(C)** Electron microscopy of synapses in the hippocampus showed that the average area of the synapse was larger in 3xTg-AD + PBS mice, which was normalized by DA4-JC. **(D)** The number of synapses was lower in 3xTg-AD + PBS mice, which was increased by DA4-JC. **(E)** Typical Golgi stain images of dendritic spines of the hippocampus in four groups. **(F)** The number of dendritic spines was less in 3xTg-AD + PBS mice, while DA4-JC prevented this reduction. Modified from Cai et al. (2021). ***p < 0.001.

**FIGURE 4:**
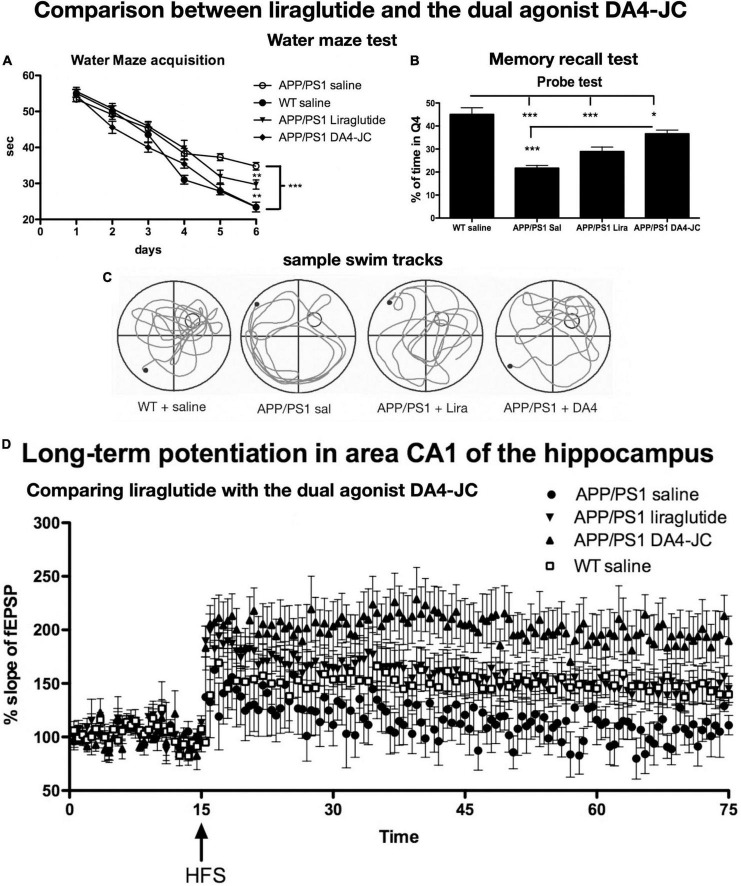
Comparison between liraglutide and DA4-JC in the APP/PS1 mouse model. **(A)** Water maze acquisition. DA4-JC facilitated learning more than liraglutide. **(B)** Recall test of the task. DA4-JC improved memory recall compared to liraglutide. *p < 0.05; **p < 0.01; ***p < 0.01. *N* = 12 per group. **(C)** Sample swim tracks of mice are shown. **(D)** LTP in area CA1 of the hippocampus is improved by DA4-JC more than by liraglutide. The saline group did not develop significant LTP. *N* = 6 per group. Modified from Maskery et al. (2020).

**FIGURE 5:**
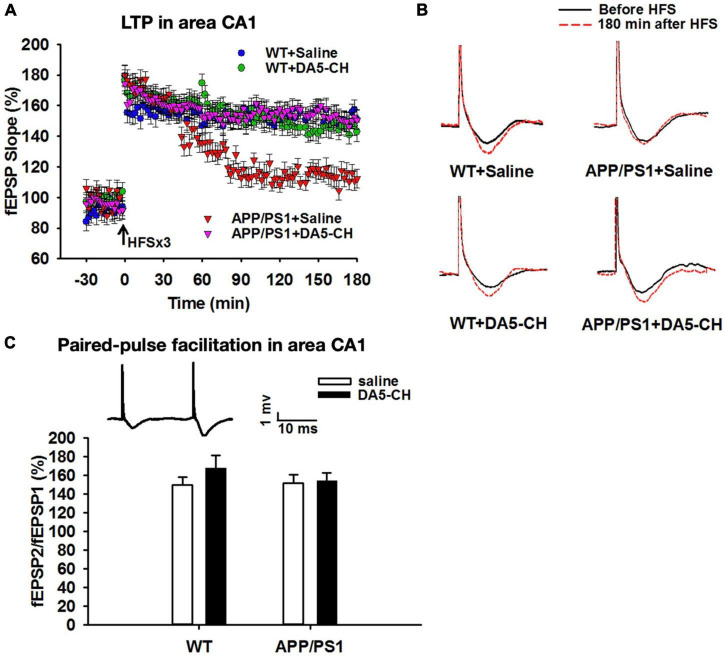
DA5-CH effectively reversed the impairment of LTP in area CA1 in APP/PS1 mice. **(A)** Scatter plots showing the changes of fEPSP slope in different groups. After the application of three sets of HFS, LTP was induced in all groups, but the maintenance of LTP was impaired in APP/PS1 + Saline mice, and responses went back to baseline. DA5-CH rescued LTP in APP/PS1 mice, comparable to DA4-JC. *N* = 6 per group. **(B)** Representative fEPSP traces before (black) and 180 min after (red) HFS. **(C)** Paired-pulse facilitation (PPF) percentage in area CA1. No change was observed at this age in this mouse model. Insets: representative PPF traces. Modified from Cao et al. (2018).

The dual-receptor agonist DA3-CH that does not have any modifications, such as acylation to increase the survival time in the blood, enters the brain easier and shows good neuroprotective effects in the APP/PS1 mouse model. DA3-CH improved learning and memory in spatial water maze tasks. Levels of amyloid plaques in the brain were reduced, and biomarkers for autophagy functionality were improved (Panagaki et al., 2018). In the 3xtg mouse model of AD, which expresses human mutated genes of APP, PS1, and tau, it was found that spatial memory was rescued by DA4-JC. Furthermore, LTP in area CA1 of the hippocampus was much improved by the drug, almost to the level of wild-type controls. Importantly, when quantifying synapse numbers in the hippocampus using electron microscopy and Golgi stain methods, it was found that the 3xtg mouse model developed loss of synapses, which was prevented by DA4-JC (Cai et al., 2021; see Figure 3 for details). In a direct comparison between the dual agonist DA4-JC and liraglutide, DA4-JC was superior in improving memory formation. When testing LTP formation in the area CA1 of the hippocampus of APP/PS1 mice, DA4-JC was superior to liraglutide in enhancing LTP and protecting hippocampal neurons from damage. LTP was almost at the same level as wild-type controls (see Figure 4). Amyloid levels were more reduced by DA4-JC than by liraglutide treatment. The chronic inflammatory response in the brain and levels of pro-inflammatory cytokines was improved by DA4-JC, more than by liraglutide (Femminella et al., 2019). In addition, we tested the dual agonist DA5-CH in the APP/PS1 AD mouse model. DA5-CH improved memory formation and lowered amyloid plaque and phosphorylated tau protein levels in the brain. In hippocampal recordings, DA5-CH reversed the impairment of LTP (Cai et al., 2021; see Figure 5 for details of this study). Furthermore, DA5-CH showed good neuroprotective effects in the icv. streptozotocin (STZ) rat model of AD. In this model, insulin signaling is much reduced in the brain by STZ, and tau phosphorylation is enhanced. After drug treatment, tau phosphorylation levels in the brain were reduced, insulin signaling normalized, and the inflammatory response in CNS was also reduced. In EEG recordings of freely moving rats, STZ icv. treatment reduced theta rhythm in the hippocampus, and DA5-CH reversed this impairment (Li et al., 2020). Table 1 lists the results of novel dual-GLP-1/GIP receptor agonists in animal models of AD.

**TABLE 1:** Overview of publications that tested GLP-1/GIP dual agonists in animal models of AD or PD.

Animal model	Drug tested	Result	References
MPTP mouse model of PD	DA1-JC	Improvement in motor tasks, protection of dopaminergic neurons, enhanced release of BDNF, Pi3k activity was enhanced, pro-apoptotic signaling reduced	Ji et al., 2016
MPTP mouse model of PD	DA1-JC	Improvement in motor tasks, protection of dopaminergic neurons, reduced inflammation in the brain, enhanced synaptic protein levels	Cao et al., 2016
SH-SY5Y cell culture treated with rotenone	DA1-JC	DA1-JC is the most effective drug compared to single GLP-1 or GIP analogs or to oxyntomodulin. Protection of cell viability, reduction of apoptotic signaling, improvement of autophagy, and Pi3k signaling	Jalewa et al., 2016
6-OHDA rat model of PD	DA1-JC	Motor activity was improved, dopaminergic neuronal loss was reduced, levels of GDNF were increased and pAkt/CREB signaling normalized	Jalewa et al., 2017
icv. STZ rat model of AD	DA4-JC	Memory formation was improved, tau phosphorylation reduced, insulin signaling improved, reduced apoptosis and inflammation in the brain	Shi et al., 2017
MPTP mouse model of PD	DA3-CH	DA3-CH is more effective than liraglutide in improving motor activity, protecting dopaminergic neurons, reducing inflammation, and levels of GDNF are increased	Yuan et al., 2017
MPTP mouse model of PD	DA1-JC, DA4-JC, DA5-CH	DA4-JC and DA5-CH are more effective in protecting the brain than liraglutide and DA1-JC. Motor activity was improved, dopaminergic neurons protected, levels of pro-inflammatory cytokines reduced, GDNF levels were increased	Feng et al., 2018
APP/PS1 mouse model of AD	DA5-CH	Memory formation was protected, synaptic plasticity (LTP) preserved and tau phosphorylation reduced, PI3k and Akt activity normalized	Cao et al., 2018
APP/PS1 mouse model of AD	DA3-CH	DA3-CH improved memory formation, normalized autophagy, reduced ER stress and apoptotic signaling, reduced amyloid plaque load in the brain	Panagaki et al., 2018
icv. Amyloid (31-35) AD model	DA1-JC	memory formation was improved, and disturbance of circadian rhythm improved	Wang et al., 2019
APP/PS1 AD mouse model	DA1-JC	DA1-JC was more effective than liraglutide in a slow-release formulation in improving memory formation, reducing inflammation, and reducing oxidative stress	Salles et al., 2020
icv. STZ rat model of AD	DA5-CH	Memory formation is rescued, EEG theta rhythm normalized, tau phosphorylation is reduced, apoptosis signaling is reduced, CREB signaling is normalized, DA5-CH is superior to DA1-JC, liraglutide or exendin-4 in crossing the blood-brain barrier	Li et al., 2020
MPTP mouse model of PD	DA5-CH	Receptor binding study of DA5-CH showing selective binding to GLP-1 and GIP receptors. DA5-CH is superior to DA1-JC, DA2, DA3-CH, liraglutide or exendin-4 in crossing the blood-brain barrier. DA5-CH is superior to exendin-4 in a dose-response study. Motor activity is protected, inflammation is reduced, lipid oxidation is reduced, apoptosis is reduced, DA5-CH is superior to liraglutide	Zhang et al., 2020
APP/PS1/Tau AD mouse model	DA4-JC	DA4-JC receptor binding study shows selective binding. DA4-JC is superior to liraglutide in a dose-response study in reducing amyloid plaques. DA4-JC was more effective than liraglutide in reversing memory loss, enhancing synaptic plasticity (LTP) in the hippocampus, reducing amyloid plaques and lowering pro-inflammatory cytokine levels in the brain.	Cai et al., 2021
6-OHDA rat model of PD	DA5-CH	DA5-CH is more effective than exendin-4 in protecting motor activity, reducing a-synuclein levels and pro-inflammatory cytokine levels in the brain. It was also more effective than exendin-4 in reducing apoptotic signaling. Insulin desensitization was reversed and the levels of autophagy markers were normalized.	Zhang et al., 2021
MPTP mouse model of PD	DA5-CH	DA5-CH is more effective than NLY01, a 40 kDa pegylated form of exendin-4, in protecting motor activity, reducing α-synuclein levels, NF-kB and pro-inflammatory cytokine levels in the brain. DA5-CH can cross the BBB better than the pegylated exendin-4 drug.	Lv et al., 2021

## Glucagon-like peptide 1, glucose-dependent insulinotropic peptide, and dual- Glucagon-like peptide 1/glucose-dependent insulinotropic peptide receptor agonists show good effects in animal models of Parkinson’s disease

PD is a chronic disease that involves the failure of dopaminergic synapses and the loss of neurons in the SN. We and others have shown that GLP-1 receptor agonists or analogs of GIP can improve synaptic transmission in the striatum, and protect synapses and neurons from PD-related stressors (Liu et al., 2015; Zhang et al., 2015; Li L. et al., 2016; Zhang and Hölscher, 2020; Hölscher, 2022). In the 1-methyl-4-phenyl 1,2,3,6-tetrahydropypridine (MPTP) mouse model of PD, such drugs showed good effects in protecting the brain from MPTP-induced toxicity. MPTP is a chemical that can induce PD in humans (Langston et al., 1983) and that shows PD-like symptoms in rodents (Bove and Perier, 2012). The synthesis of dopamine by neurons and the levels of dopamine in the striatum are much reduced by MPTP, demonstrating that synaptic transmission is impaired (Verma et al., 2017). We were able to show that the synaptic marker synaptophysin was reduced in mice treated with MPTP, and that a GIP analog was able to increase the levels (Li Y. et al., 2016). Dopamine levels in the striatum of MPTP-treated mice were reduced, and a GIP analog was able to reverse and normalize the levels (Verma et al., 2017). Dual-GLP-1/GIP receptor agonists have been effective in protecting mice from MPTP toxicity. When comparing liraglutide with several dual agonists, it was found that the dual agonists that can cross the BBB better were superior to liraglutide. DA4-JC and DA5-CH, dual agonists that contain a cell penetrating peptide (CPP) motif that increases their ability to cross the BBB, were better than dual agonists that do not contain a CPP (Feng et al., 2018; Zhang et al., 2020; see Figure 1 for details). In the 6-OHDA rat model of PD, we were able to show that a dual-GLP-1/GIP receptor agonist can rescue dopaminergic neurons from the toxin and can increase dopamine levels in the striatum (Jalewa et al., 2017). Table 1 lists the results of novel dual-GLP-1/GIP receptor agonists in animal models of PD.

## The Glucagon-like peptide 1 receptor agonists exenatide and liraglutide show good effects in clinical trials

Building on the protective effects of GLP-1 receptor agonists that we and others have shown, four clinical trials have been conducted, testing the GLP-1 receptor agonists exendin-4 or liraglutide. Both drugs are on the market as treatments for type 2 diabetes. Changes in GLP-1 signaling have been observed in the brains of AD patients, with reduced expression of GLP-1 receptors but retained functionality of GLP-1 signaling in the brain (Talbot et al., 2011). An analysis of GLP-1R mRNA in patients with PD showed a 10-fold increase in the SN (Yun et al., 2018), indicating that GLP-1 levels in the brain are reduced, which leads to an upregulation of the receptor. First clinical trials have shown that GLP-1 receptor agonists can improve clinical pathologies of PD and AD, including prolonged benefits on cognition and motor symptoms, and are a proof of concept (Aviles-Olmos et al., 2013, 2014; Athauda et al., 2017, 2019a; Athauda and Foltynie, 2018; Edison et al., 2021).

### Exendin-4 in Parkinson’s disease

In a randomized, single-blind, open-label pilot study (NCT01174810), 45 subjects with moderate PD who were on standard L-Dopa-based treatment were allocated to either exendin-4 (Byetta) twice daily treatment for 12 months or were controls without treatment. The 12-month treatment period was followed by a 2-month “drug wash-out” period to compare exendin-4 with a control group without the drug. After 14 months, the result showed significant differences in both cognitive and motor measures: a 6.3-point improvement in Mattis dementia rating scale-2 (Mattis DRS-2) and a 7.2-point improvement in the Movement Disorders Society Unified PD Rating Scale (MDS-UPDRS) part 3 in the drug group compared to controls (Aviles-Olmos et al., 2013). Exendin-4 was well tolerated by the patients, with minor nausea and weight loss being common side effects that were considered non-critical. The motor test battery MDS-UPDRS part 3 was conducted by Neurologists blind to the treatment. The benefits in both motor and cognitive behaviors in the drug group were still visible in a follow-up assessment after a “drug wash-out” phase of 12 months after the last drug treatment (Aviles-Olmos et al., 2014). This demonstrates that the improvements in motor control and cognitive tests are not simply a placebo effect.

These encouraging results motivated a follow-up phase II clinical trial that had a placebo control group. The once-weekly formulation of exendin-4 (Bydureon) was tested in PD patients for 60 weeks by the same investigators. In this randomized, double-blind, and placebo-controlled phase II clinical study (NCT01971242) (Athauda et al., 2017), 60 patients with moderate PD were randomized to subcutaneous administration of exenatide (Bydureon; once weekly) or placebo for 48 weeks in addition to their dopaminergic replacement drugs, directly followed by a “wash-out” period of 12-weeks. The primary outcome of the trial was the severity of PD motor signs assessed by part 3 of MDS-UPDRS after 60 weeks and in the off-medication state. Patients that had received the drug showed much better values in the motor tests than the placebo group. Following 48 weeks of exenatide therapy, the adjusted between-group difference in part 3 of MDS-UPDRS was 4.3 points. At 60 weeks, off-medication scores in part 3 of MDS-UPDRS had improved by 1.0 points in the exenatide group while had deteriorated by 2.1 points in the placebo group, and adjusted mean difference of –3.5 points. Cerebrospinal fluid (CSF) analysis demonstrated that exendin-4 can cross the BBB, and furthermore showed that it was no longer present after wash-out (Athauda et al., 2017). The improvements in MDS-UPDRS part 3 scores were still visible after “wash-out” at 60 weeks, demonstrating that the drug effect was still there after the drug had gone. This clearly shows that the effects of exendin-4 last beyond the acute drug effect, satisfying the definition of disease-modifying agents. In addition, a range of additional secondary measures was assessed, most of which showed an improvement, though they did not show statistical significance after multiple comparison adjustments (Foltynie and Athauda, 2020).

A *post hoc* analysis of this clinical trial that evaluated the non-motor PD-related symptoms, such as mood/apathy, cognition, and memory, found that some of the symptoms improved in exendin-4-treated patients, though these benefits did not always persist after drug delivery stopped (Athauda and Foltynie, 2018). Another *post hoc* analysis indicates that PD patients with obesity may have a better cognitive response to exendin-4 compared with other subgroups of PD patients (Athauda et al., 2019b). Exosomes are vesicles that are released from cells. Neuronal exosomes taken from blood samples from patients that took part in this clinical trial showed improvements in insulin signaling in the brain, as predicted by GLP-1 drug effects in diabetes. It was found that standard biomarkers of insulin signaling, such as IRS-1 phosphorylation at tyrosine sites, was improved in the brains of PD patients in comparison to patients who received a placebo (Athauda et al., 2019a).

### Liraglutide in Parkinson’s disease

Another phase II clinical trial that was randomized and double-blind showed that liraglutide could improve PD pathology (NCT02953665). PD patients received once-daily liraglutide injections for 52 weeks while maintaining standard drug treatments, such as L-Dopa. The primary analysis included 37 drug-treated and 18 placebo patients. After 54 weeks of treatment, the non-motor symptom scores (NMSS) had improved by 6.6 points in the drug group, but got worse by 6.5 points in the placebo group—a 13.1-point adjusted mean difference in total. The difference just missed statistical significance (*p* = 0.07). The MDS-UPDRS part III and MDRS-2 scores improved but were not significantly different between groups, most likely due to a placebo effect in the control group. Importantly, a significant improvement in the MDS-UPDRS part-II evaluation was found in the liraglutide group (–4.1 points, *p* = 0.001). This test battery evaluates everyday activities, such as walking, talking, eating, getting dressed, getting out of a car or of a deep chair, tremor, and more (Malatt et al., 2022). In addition, global MDS-UPDRS and PDQ-39 (quality of life) scores were also significant. The result clearly documents that liraglutide improved the daily life of PD patients beyond the effects of L-Dopa. Considering the small size of this trial, the results are impressive and are a testament to the effectiveness of GLP-1 receptor agonists in PD.

### Liraglutide in Alzheimer’s disease

A pilot study testing liraglutide in a small cohort of AD patients showed some initial effects (NCT01469351). In this 26-week trial, 38 AD patients were randomized to treatment with liraglutide (*n* = 18) or placebo (*n* = 20). Due to the small number of patients, assessment of cognitive improvement was underpowered but did show a trend toward improvement. An ^18^FDG-PET brain scan showed an improvement in the PET signal, though, which is interpreted as an improvement of glucose uptake into neurons (Gejl et al., 2016).

We have conducted a placebo-controlled double-blind phase II clinical trial testing liraglutide in 200 AD patients (the ELAD study, NCT01843075). Liraglutide was given for 12 months. The trial measured cognition (ADAScog and exec), ^18^FDG-PET activity, and brain volume changes as measured by MRI brain scans, with other parameters, such as the content of exosomes that originate from the brain still being analyzed (Femminella et al., 2019, 2020). It was found that liraglutide reduced cognitive impairments in the ADASexec test battery (*p* < 0.01). Importantly, we found that brain temporal lobe volumes shrank less in the liraglutide group compared to the placebo group (*p* < 0.001), and the total gray matter cortical volume shrank less, as shown in MRI brain scans (*p* = 0.002). This suggests that neuronal loss in the brain has been reduced by liraglutide. Other AD biomarkers have not been analyzed yet (Edison et al., 2020, 2021). The result demonstrates that GLP-1 receptor agonists are not only effective in PD patients but also in AD patients.

### Ongoing clinical trials

Other randomized, double-blind, and placebo-controlled trials of GLP-1 RAs in PD patients are currently underway,^1^ testing the agents semaglutide (NCT03659682), liraglutide (NCT02953665), lixisenatide (NCT03439943), NLY01 (NCT04154072), or PT320 (NCT04269642) in PD patients, with additional phase 2 (NCT04305002) and phase 3 (NCT04232969) trial testing exenatide in PD patients in recruiting, highlighting the growing demand for discovery and development of GLP-1 RAs. Two-phase III clinical trial testing semaglutide in AD patients are currently underway (NCT04777396 and NCT04777409).

## Conclusion

A key feature of diseases, such as AD and PD, is the loss of synaptic transmission followed by loss of synapses (Davies et al., 1987; Picconi et al., 2005; Bagetta et al., 2010). Information processing and memory formation are dependent on functional synaptic transmission in AD, and the initiation of motor programs in the striatum is dependent on dopaminergic synaptic transmission in PD. Treatment strategies for these diseases should focus on keeping synaptic activity intact and preventing the loss of synapses. We have introduced a viable strategy to improve synaptic transmission and LTP in AD, as well as dopaminergic transmission in PD by the use of growth factors that can cross the BBB. We presented preclinical data that show the beneficial effects of GLP-1 and GIP receptor agonists that have been designed to cross the BBB. First clinical trials have shown encouraging neuroprotective effects in AD and PD patients, testing older GLP-1 receptor agonists that have been designed to treat type 2 diabetes. The results are proof of principle that demonstrates that this strategy is successful. The future looks bright for the development of effective and safe treatments for these chronic neurodegenerative disorders for which there are currently no disease-modifying drugs available.

## Author contributions

The author confirms being the sole contributor of this work and has approved it for publication.

## Footnotes

1
http://www.clinicaltrials.gov
